# Linking bacteria, volatiles and insects on carrion: the role of temporal and spatial factors regulating inter-kingdom communication via volatiles

**DOI:** 10.1098/rsos.220555

**Published:** 2022-08-31

**Authors:** Christian von Hoermann, Sandra Weithmann, Johannes Sikorski, Omer Nevo, Krzysztof Szpila, Andrzej Grzywacz, Jan-Eric Grunwald, Frank Reckel, Jörg Overmann, Sandra Steiger, Manfred Ayasse

**Affiliations:** ^1^ Department of Conservation and Research, Bavarian Forest National Park, Grafenau, Germany; ^2^ Institute of Evolutionary Ecology and Conservation Genomics, University of Ulm, Ulm, Germany; ^3^ Department of Microbial Ecology and Diversity Research, Leibniz Institute DSMZ-German Collection of Microorganisms and Cell Cultures GmbH, Braunschweig, Germany; ^4^ German Centre for Integrative Biodiversity Research (iDiv) Halle-Jena-Leipzig, Leipzig, Germany; ^5^ Institute of Biodiversity, Friedrich Schiller University, Jena, Germany; ^6^ Department of Ecology and Biogeography, Nicolaus Copernicus University, Torun, Poland; ^7^ Bavarian State Criminal Police Office, SG 204, Microtraces/Biology, 80636 Munich, Germany; ^8^ Department of Evolutionary Animal Ecology, University of Bayreuth, Bayreuth, Germany

**Keywords:** carrion-associated flies, community ecology, carrion ecology, epinecrotic bacteria, inter-kingdom interdependence, volatile organic compounds (VOCs)

## Abstract

Multi-kingdom community complexity and the chemically mediated dynamics between bacteria and insects have recently received increased attention in carrion research. However, the strength of these inter-kingdom interactions and the factors that regulate them are poorly studied. We used 75 piglet cadavers across three forest regions to survey the relationship between three actors (epinecrotic bacteria, volatile organic compounds (VOCs) and flies) during the first 4 days of decomposition and the factors that regulate this interdependence. The results showed a dynamic bacterial change during decomposition (temperature–time index) and across the forest management gradient, but not between regions. Similarly, VOC emission was dynamic across a temperature–time index and the forest management gradient but did not differ between regions. However, fly occurrence was dynamic across both space and time. The strong interdependence between the three actors was mainly regulated by the temperature–time index and the study regions, thereby revealing regulation at temporal and spatial scales. Additionally, the actor interdependence was stable across a gradient of forest management intensity. By combining different actors of decomposition, we have expanded our knowledge of the holistic mechanisms regulating carrion community dynamics and inter-kingdom interactions, an important precondition for better describing food web dynamics and entire ecosystem functions.

## 1. Introduction

Carcass research traditionally focuses on individual disciplines (e.g. bacteriology, chemistry or entomology) for an understanding of the variations in the decomposition process. However, in terrestrial ecosystems, vertebrate carrion is a focal point for complex multi-species communities that continually interact with each other during decomposition [1–4] resulting in the pulsed release of carrion nutrients into the surrounding ecosystem [5]. In order to develop a holistic, mechanistic understanding of the way that ecological factors regulate the biotic inter-kingdom community dynamics taking place during decomposition, an integrative research in carrion ecology is needed that connects the various research fields [3,6–12].

In terrestrial ecosystems, microorganisms are important drivers initiating the decomposition process of vertebrate cadavers [2,13–15]. The cadaver-associated bacterial communities comprise species from the host itself, from the surrounding soil and they are introduced by invertebrate or vertebrate scavengers [4,16]. Bacterial communities break down macromolecules of cadaver tissue while releasing volatile organic compounds (VOCs) as by-products of the degradation process [17–19]. These volatiles act as olfactory cues for the attraction of primary colonizers such as flies (e.g. Diptera: Calliphoridae, Muscidae and Sarcophagidae) that use the resource for nutrition as adults and larval stages or as a reproduction site [7,20–25]. As decomposition of the cadaver progresses, changes in the bacterial community are expected to result in altered volatile profiles and thus indirectly in successional arrivals of other insect taxa, e.g. beetles [26–29]. Together, all carrion-associated bacterial and eukaryotic species, the so-called ‘necrobiome’, interact with the carcass and its surroundings, and, in interaction, modify the carrion substrate resulting in its complete decomposition over time [2,30].

At the start of decomposition, bacterial communities directly determine the amount and composition of VOCs via the production of metabolites of the decay process [31] and, thus, indirectly regulate the attraction of flies towards carrion [7,23,32]. The characterization of bacterial communities over decomposition time is complex, but molecular culture-independent techniques, such has next-generation sequencing, raise new options to better approaches to the complete bacterial diversity occurring on carrion [2,4,15,33–38]. A general shift from internal aerobic to anaerobic bacterial taxa is described before internal (endonecrotic) bacteria encounter external (epinecrotic) bacterial taxa [35,39,40]. Furthermore, a shift from diverse epinecrotic bacterial communities during the beginning of decay to more similar communities over the course of time has been reported [14,19,41]. At the start of decomposition, epinecrotic bacterial communities resemble the host microbiome and, with progressing decay, evolve to a community that is more similar and homogenized to the surrounding environmental microbiome [13–15,38,42].

In addition to VOC production by bacterial degradation, the VOCs that are emitted from carrion can also be released from the carrion itself (e.g. from the gastrointestinal tract), from secretions of insect larval aggregations, and from invertebrate and vertebrate scavengers that bring their own odour profiles [43–45]. At the start and the end of decomposition, smaller amounts and numbers of various volatiles are released, whereas most complex odour blends are emitted in the active phases of decay (volatile-enriched liquids leaking out from the body) [27,46,47].

Within minutes or hours of death, adult female dipterans, such as blow flies and flesh flies, locate carrion by using carrion volatiles, such as dimethyl disulfide, phenol and indole, as olfactory cues to find optimal resource conditions for oviposition [22,48]. As decomposition progresses, odours from secretions of hatched fly larvae feeding on the cadaver in turn attract adult conspecifics [43,49,50]. Therefore, the highest fly abundance and species numbers are found early in the decomposition process [51–53]. In their turn, masses of arriving flies act as vectors by transmitting their own microbiome, internally by excretion or regurgitation and externally by microbial footprints, which are most likely associated with environmental microbes [54–58]. Additionally, immature and mature insects aggregating on carrion release antimicrobial secretions and hence might also influence the bacterial community composition on cadavers [59–63]. Moreover, soil microorganisms that are in contact with the carrion substrate partly determine the decomposer microbiome [16,64]. Everything considered together, inter-kingdom interplay is apparent wherever bacterial assemblages influence insect assemblages on decomposing remains and vice versa; this is especially true within the first days of decomposition, but the mechanisms and ecological consequences behind such interplay are poorly understood [1–4].

Because of the multi-trophic communication occurring on carrion, changes of bacterial compositions and thus volatile profiles might have cascading effects on the colonization patterns of insects by shaping their abundance, richness and community structures [8,25]. Two-way relationships of different carrion-associated actors (between bacteria and volatiles, volatiles and flies, or bacteria and flies) have been addressed in several studies [19,20,22,25,65–68]. Especially at the beginning of the decomposition, bacteria and carrion-associated flies strongly interact with each other and significantly contribute to the modification of the carcass [4]. By contrast, tritrophic bacteria–volatile–insect interactions in a natural environment have been little examined. Laboratory experiments have contributed to an increasing understanding of such inter-kingdom relationships at the species level [66,69,70] and underline the ecological importance of the complex interdependence between these three actors during decomposition [4]. However, the underlying mechanisms that drive three-way inter-kingdom interactions at the community level and during natural decomposition processes are poorly known [1–3].

Not only the initial state of the cadaver itself and its physiological and morphological post-mortem changes through time, but also the surrounding habitat conditions cause diverse responses of the biotic communities. Ambient temperature is the main environmental driver determining the physiological and chemical reactions of the vertebrate decomposition process [8,71–76] and a positive relationship between ambient air temperature and responses of several actors on carrion is evident: bacterial activity on carrion changes with higher temperatures [15,72], as does VOC production and emission [31,47], the immature insect growth rate (e.g. [77]), and adult insect activity and oviposition behaviour [51,74,76,78–80]. Hence, temperature variations on site might also regulate inter-kingdom communications taking place on the carrion resource. Furthermore, spatial environmental gradients such as increasing forest management or habitat type variations limit the conditions for insect communities to locate randomly occurring carrion [81,82]. Additionally, increasing management activities in German forest stands ranging from near-natural forests to monocultures shape the communities of the local carrion-associated rove beetles (Coleoptera: Staphylinidae) by promoting generalist species that can cope with, for example, low structural heterogeneity [82]. Therefore, the likelihood increases that more generalist beetle species are able to locate the carrion in such forest sites. However, with regard to carrion-associated flies, their abundance has been found to be higher in forest fragments than in contiguous forests in a rural–urban gradient near New York, USA [83], and more individuals and species of flies have been found in German forests having a lower canopy cover [84]. Concerning the bacterial component of the necrobiome, current studies indicate that human-induced forest management plays a minor role in shaping bacterial communities in forest soils, whereas the soil pH mainly determines changes in these soil bacterial compositions [85]. Soil microorganisms mainly encounter the internal microbiome of a carcass after the body opens because of the overpressure of bacterial gases. This process is expected substantially to affect bacterial structures but not before progressed decay [6]. However, epinecrotic bacterial communities might be influenced earlier in the decomposition process, because insects, walking on the surface and within the orifices of the cadaver, bring their own microbiome from potentially anthropogenic altered soils and habitats [6,21,55]. Another important aspect regarding the modification of inter-kingdom interactions at the carrion resource is the spatial geographic distribution of carrion-associated species. It determines which insect species is able to colonize randomly occurring carrion, e.g. regional prevailing species pools of rove beetles [82], dung beetles [86] and carrion-associated flies [87]. These examples demonstrate that environmental gradients have to be considered in comprehensive carrion ecological assessments with regard to their potential modifications of inter-kingdom interactions at the carrion resource [6]. An understanding of the way which environmental abiotic factors regulate community networks might be important for describing the variation of biotic assemblies on carrion under different conditions and the resulting variation in decomposition speed between individual carcasses [11,12,70,88].

The purpose of the present study is twofold. In the first part, we evaluate the temporal and spatial factors that shape the abundance, richness, composition and species turnover of each separate actor (bacteria, volatiles and flies) collected on 75 stillborn piglet cadavers during the whole aboveground decomposition process. In the second part, we investigate the strength of interdependence between bacteria, volatiles and flies at the community level and aim to identify the underlying mechanisms regulating this interdependence. We have specifically asked the following questions:

— How do temporal and spatial factors affect the alpha-diversity (total abundance and species richness) and beta-diversity (community composition and species turnover) of each separate actor (bacteria, volatiles and flies) on carrion?
— Do the three actors show an interdependent relationship?
— How do temporal and spatial factors regulate the interdependence of the three actors?

## 2. Material and methods

### 2.1. Study sites and piglet cadaver exposure

This study was performed in the framework of the interdisciplinary priority programme ‘the Biodiversity Exploratories' (http://www.biodiversity-exploratories.de) [89]. We conducted the fieldwork in the three study regions of the Biodiversity Exploratories in Germany: the Biosphere Reserve Schorfheide-Chorin (SCH; German state of Brandenburg) in the northeast, the National Park Hainich-Dün (HAI; German state of Thuringia) in the middle and the Biosphere Reserve Schwäbische Alb (ALB; German state of Baden-Württemberg) in the southwest of the country. Seventy-five experimental forest sites (25 sites for each study region) representing forest stands along a gradient of forest management intensity were selected, ranging from near-natural mixed forest stands (dominated by beech) to highly managed conifer forests (mainly spruce and pine). All 75 forest experimental plots of one hectare each were selected following a stratified random design with strata representing diverse intensities of forest management and several other abiotic factors like soil depth and soil type (fig. A1 in [86,89]). The intensity of forest management was given by a precalculated silvicultural management intensity (SMI) index for each forest stand and was based on three main criteria of a wooden stand: the main tree species, the stand age, and the amount of living and dead woody aboveground biomass [90]. The SMI index is a numerical indicator of how intensively a forest stand is managed by humans under natural conditions (range from 0: near-natural stands up to 1: clear-cut). For a more detailed description of the prevailing habitat parameters of each experimental forest site like vegetation cover, main tree species, soil type etc., see electronic supplementary material, table S3 (variables and their respective values were obtained from the BExIS platform, Biodiversity Exploratories Information System, https://www.bexis.uni-jena.de; associated detailed data source information is provided in the electronic supplementary material, table S4).

For our experiment, we used 75 stillborn piglet (*Sus scrofa domestica*) cadavers (1.44 kg average weight, min. of 1.2 kg, max. of 2.3 kg) as standardized carrion substrate. All piglets were purchased from one local farmer (same food substrate for all mother sows) and were immediately frozen at −20°C upon stillborn birth. The collection effort of stillborn piglets from the pig breeder farm lasted from 26 May 2014 (the first 11 cadavers), 30 May 2014 (32 cadavers) up to 27 June 2014 (the last 32 cadavers). Consequently, the 75 cadavers were not frozen the same day and during the same period. Freezing of vertebrate tissues is reported to slightly bias the process of decomposition by favouring aerobic decay over putrefaction (anaerobic process) during the first days of decomposition [91]. For minimizing such a potential impact on later data interpretation, we restricted the total collection time of overall 75 piglet cadavers to one month. In August 2014, we simultaneously exposed the cadavers aboveground on each of the 75 experimental forest sites (one piglet per site) in wire cages (63 cm × 48 cm × 54 cm, MH Handel GmbH, Munich, Germany). Data loggers (Thermochron iButton, Whitewater, WI, USA) installed in the top corner of each wire cage recorded ambient air temperature every 30 min for the whole decomposition time (the temperature profiles during the whole study period are illustrated in electronic supplementary material, figure S1). We visited the piglet cadavers at regular inspections on days 2, 4, 6, 9, 16, 23 and 30 days after exposure on day 0 and sampled material (bacteria, volatiles and insects) in parallel (see below). During each sampling event, photographs were taken to assess and document the decomposition stage of the cadavers based on the visual criteria adapted after Payne [80]. Thereby, six different stages of decomposition as typical for terrestrial aboveground decomposition were described: fresh, putrefaction, bloated (conspicuous appearance of putrefaction), post-bloating, advanced decay and dry remains. Because the anaerobic bloated appearance of a carcass might be not visible or skipped in some cases, we described the anaerobic putrefaction stage as an important existing stage between the fresh and the aerobic post-bloating stages. In a study examining piglet carrion inhabiting bacteria, Pascual *et al*. [19] successfully described the function of bacterial community dynamics in the formation of cadaveric semiochemicals during carcass decomposition by evaluating six different decomposition stages, including the putrefaction stage.

### 2.2. Bacterial sampling and 16S rRNA gene sequencing analysis

We sampled bacteria from the snout mucosae via sterile cotton swabs (Copan FLOQSwabs™, MAST Diagnostica GmbH, Reinfeld, Germany) by using a reproducible and quantifiable swab protocol (three times swabbing in the upper and three times swabbing in the lower part in the piglet snout). The snout mucosae were chosen because Pechal *et al*. [92] documented that most microbial community variability over decomposition occurred in the mouth. Bacteria were sampled as long as snout mucosae were clearly detectable on days 0, 2 and 4 during piglet decomposition. Immediately after sampling, the swabs were stored in sterile tubes filled with a buffer reagent (RNAlater^®^, Sigma-Aldrich, Germany) and were frozen at −80°C until further processing.

We extracted bacterial DNA from the swab samples by using a DNeasy Blood & Tissue kit (Qiagen, Crawley, UK) following the manufacturer's protocol. For the polymerase chain reaction, we used a two-step amplification procedure after Bartram *et al*. [93] targeting the V3 region of the prokaryotic 16S rRNA gene and employing primers and conditions described as in Pascual *et al*. [19]. The amplification products were sequenced with the Illumina Nextseq 500 High Throughput Sequencing System (Illumina, San Diego, CA, USA).

Raw sequence reads were processed using plugins available in the QIIME 2^TM^ platform (https://qiime2.org/, v. 2017.12) [94]. Raw sequence reads were imported, joined, quality filtered, chimera-checked and denoised, and the obtained distinct sequences were subsequently allocated to samples by using the plugins *vsearch*, *quality filter* and *deblur* [95,96]. The taxonomic classification of representative sequences was performed using a naive Bayes classifier that was trained using the 16S rRNA gene sequence database of SILVA (version 128) [97] using the plugin *feature-classifier*. For further statistical analysis, the read abundance table of exact sequence variants (SVs) [98] and the taxonomic classification were imported into the R package *phyloseq* v. 1.24 [99]. Coverage-based rarefaction on the level of SVs was carried out using the function *phyloseq_coverage_raref* in R package *metagMisc* [100] which aimed to rarefy all samples to a sample coverage of 0.999. The overall high sample coverage (an index for the level of completeness of a sample) of 0.999 (which is the minimum value across sites) indicates that samples have been sequenced to saturation depth, and rarefication to same sample coverage has been shown to be ecologically more informative than rarefication to same read count size [101].

### 2.3. Cadaver volatiles sampling and chemical analyses

VOCs that were emitted during decomposition were collected at sampling days 2, 4, 6, 9, 16, 23 and 30 via the dynamic headspace adsorption technique, adapted after [102]. For that purpose, we placed vacuum pumps (DC12, Fürgut, Tannheim, Germany) in front of the piglet abdomen and pumped off the surrounding air for 5 min through a ChromatoProbe filter system filled with an adsorbent material (1.5 mg Tenax^®^ and 1.5 mg Carbotrap^®^, Supelco, Bellefonte, PA, USA) (electronic supplementary material, figure S3). The incoming air (flow rate of 500 ml min^−1^) passed through the precleaned filters, and volatiles were trapped in the adsorbent material. This sampling protocol (highly sensitive ChromatoProbe adsorbent material, sampling time of 5 min as well as flow rate of 500 ml min^−1^) proved to be effective for correlating individual cadaveric VOCs with specific taxa during the first stages of decomposition which are dominated by bacteria [19]. We used the samples from the control sites (100 m from each carcass and by visual inspection free from any other decaying animal material) from day 2 as a surrogate for the initial decomposition VOCs of day 0. The filters were kept in a cooler directly after collection and were afterwards stored in a freezer at −20°C until chemical analysis.

The volatile compounds were identified by gas chromatography coupled with mass spectrometry (Agilent 7890B GC plus 5977A MSD, Agilent Technologies, Santa Clara, CA, USA) equipped with a thermal desorption unit (Gerstel, Mühlheim a. d. Ruhr, Germany) with a cold-injection system (CIS 4C; Gerstel, Mühlheim a. d. Ruhr, Germany) and a polar DB-WAX column. Detailed information about the chemical methodology can be found in Pascual *et al*. [19]. Absolute amounts (in ng) of all compounds were estimated by using Agilent ChemStation (Agilent Technologies, Germany) with an external standard (tridecane; peak area = 10 ng) as a reference. Tridecane was chosen as an external standard because this compound was not listed in the comprehensive cadaveric volatile list (832 VOCs released by a decaying pig carcass; GCxGC-TOFMS technique) in Dekeirsschieter *et al*. [103]. We employed a target compound approach by using a reference list of 43 well-known compounds emitted from carrion; this list had been built up in a preliminary study with a smaller dataset (nine cadavers from the study region SCH, published in [19]). We compared the list to the complete chemical dataset from the 75 decomposing piglet cadavers. We have chosen the targeted analysis as the preferred approach instead of a non-targeted analysis because it provides improved selectivity and sensitivity over the less specific option of scanning all compounds [104]. The reference compound list represented common volatiles found on vertebrate cadavers [103] and compounds evoking antennal receptor reactions in carrion-associated insects [28,105]. Compounds were then identified based on their mass spectra using multiple references from the NIST11 library and on published Kováts retention indices in the software Amdis 2.71 (Automated Mass Spectral Deconvolution and Identification System). Retention indices were calculated by using the following *n*-alkane reference blend: undecane (C11), dodecane (C12), tridecane (C13), tetradecane (C14), pentadecane (C15), hexadecane (C16), heptadecane (C17), octadecane (C18), nonadecane (C19), eicosane (C20), heneicosane (C21), docosane (C22), tricosane (C23), tetracosane (C24), pentacosane (C25) and hexacosane (C26). Finally, the compounds from the reference list were verified by means of strong (stronger compared with environmental controls) and abundant (minimally abundant in five samples) signals, and thus the calculated relative amounts of 22 compounds were included in further statistical analysis (figure 2). For the calculation of the relative composition of every VOC, we compared total ion abundances (semi-quantification) of each cadaveric VOC with the summed total ion abundances of all other cadaveric VOCs in a headspace sample [106].

### 2.4. Diptera sampling and identification

Close to each exposed piglet cadaver, we installed two pitfall traps filled with a scentless detergent/water solution for trapping of cadaver-associated insects for each 48 h interval (electronic supplementary material, figure S4). In carrion ecology, pitfall traps proved to be effective in capturing and mirroring a high diversity of the carrion-inhabiting community (e.g. [107,108]). In the near-natural forest of the Bavarian Forest National Park in Germany a high number of 97 necrophagous Diptera species retrieved from pitfall traps close to wildlife carcasses were confirmed by DNA metabarcoding (von Hoermann *et al*., unpublished result). In this study, we focused on four dipteran families: blow flies (Diptera: Calliphoridae), flesh flies (Diptera: Sarcophagidae), muscid flies (Diptera: Muscidae) and fanniid flies (Diptera: Fanniidae), since these insects are known as primary cadaver colonizers and are therefore, in addition to bacteria, considered as the main drivers of early vertebrate decomposition [53]. Because bacteria from detectable snout tissue were sampled during the first 4 days of decomposition (see §2.2), we focused on the associated very early carrion-inhabiting dipteran families by not taking into account the later arriving necrophagous and often on fly offspring predaceous forest beetle taxa like Staphylinidae or Silphidae [29,82,86]. Individuals of all mentioned fly families were determined to species level. Flies were identified according to the keys and publications by [109–114]. The family Calliphoridae in the analyses was treated in a broad sense, including species representing the recently established family Polleniidae [115].

### 2.5. Statistical analyses

#### 2.5.1. Data exploration and selection of response and explanatory variables

We aimed to investigate the three main actors that play important roles in the early decomposition of vertebrate tissue: epinecrotic bacteria, VOCs emitted from the carrion substrate, and carrion-associated flies (families Calliphoridae, Sarcophagidae, Muscidae and Fanniidae). Thus, the three actors were included as response variables in the statistical models.

Aboveground decomposition processes and all associated reactions of biotic and abiotic actors are highly temperature dependent. Therefore, temperature was included as a covariate into all models. However, data exploration revealed strong negative collinearity of daily mean temperature and sampling days within the first days of our study (see electronic supplementary material, figure S5). In order to avoid violating the assumptions of low collinearity between explanatory variables in the regression models [116,117], we converted time and temperature into a continuous time variable taking daily mean temperature variances between study sites into account: the temperature–time index accumulated degree-day (ADD) over a temperature threshold of 0°C (further named as ADD_0_). ADD accounts for temperature variation over decomposition time and is a reliable and frequently used predictor variable, e.g. in forensic entomological assessments in insect growth models and post-mortem interval estimations [56,78,118,119]. ADD implies the accumulation of thermal energy that is needed for biological and chemical reactions over time and is an index that thermally standardizes the progress of decomposition [30]. The formula used is as follows: DD = [{*T*_min_ + *T*_max_}/2] − *i*, where DD is degree-day, *T*_min_ and *T*_max_ are the minimum and maximum air temperatures reached at a day, and *i* is the minimum temperature threshold over which the accumulation is considered [118]. With regard to the minimum threshold, the minimum temperature for flight activity ranges in, for example, blowflies from 0°C for *Calliphora vicina* and up to 10°C for other species of Calliphoridae (*Lucilia* sp.) [78], and the minimum growth temperature for some bacteria is expected to be at 0°C [38]. Therefore, in order to create a temperature–time index useful at inter-kingdom scale (to compare bacteria, volatiles and flies), we defined the threshold value at 0°C. The use of ‘ADD_0_’ instead of ‘sampling day + daily mean temperature’ in our models has the following advantages: low correlation between all explanatory variables (electronic supplementary material, figure S5b) but the inclusion of temperature differences between sites and the creation of a continuous (instead of a categorial) scale that can be well analysed via (non)linear models, because insects are assumed to show no distinct breakpoint in their occurrence across decomposition time [118]. The range of the calculated temperature–time index ADD_0_ across the study period is shown in the electronic supplementary material, figure S6. We also decided not to compare actors between decomposition stages (as a further frequently used predictor for the temporal decomposition progress) because decomposition rates were different in all cadavers (electronic supplementary material, figure S2), and we thereby avoided the problem of pseudoreplication in the dataset. Together, ADD_0_, forest management intensity and study regions represented the three explanatory variables on the temporal and spatial scales for all further analysis. Finally, the continuous explanatory variables ADD_0_ and forest management (SMI index) included in the models were transformed using the function ((*x* – mean(*x*))/standard deviation(*x*) to normalize data mean values between 0 and 1 [120].

#### 2.5.2. Factors shaping the alpha- and beta-diversity of the three actors (bacteria, volatiles or flies)

All data were statistically analysed in R v. 4.0.4 (2021-02-15) [121] in the RStudio environment (v. 1.4.1103). In the first part of the analysis, we investigated the effect of ADD_0_, forest management intensity, and study regions on the alpha-diversity (total abundance and species richness) and beta-diversity (Bray–Curtis and Jaccard dissimilarities) of each separate actor (bacteria, volatiles or flies). For alpha-diversity estimates, total abundance was calculated using *rowSums*() in R package *base* [121] (except for the bacteria dataset, as sequence read counts are not a valid proxy for the abundance of individual specimen) and species richness using *specnumber*() in R package *vegan* [122]. Because of the nature of the nonlinear relationship between ADD_0_ and insects [123,124], the effects on alpha-diversities were quantified using generalized additive mixed effect models (GAMMs; functions *gam*() in R package *mgcv* [125,126] and *gamlss*() in R package *gamlss* [127]) with a restricted maximum-likelihood and by using various distributions (electronic supplementary material, table S1) based on the results for the diagnosis plots of the models. Piglet ID was included as a random factor in all models to constrain the variance within one piglet individual. In the GAMMs, ADD_0_ was included as a nonlinear smoothing function. The model structure was specified as response variable ∼ ADD_0_ + forest management + region + (random | piglet ID). For GAMMs calculated in the *mgcv* package, pairwise comparisons of the categorial variable region were calculated using the function *emmeans*() in the R package *emmeans* [128]. Currently, a pairwise comparison test is not yet available for the *gamlss* package. Nonlinear model fits were validated by using the function *gam.check*() in R package *mgcv* and to check the diagnosis plots. Marginal effect plots of all (non)linear models were visualized in the R package *sjPlot* [129]. The integration of confidence intervals calculated from GAMMs of the *gamlss* package into the marginal effect plots and created within the *sjPlot* package is currently not yet available.

Species compositions using Bray–Curtis dissimilarities of Hellinger-transformed relative abundance data [130] and species turnover using Jaccard dissimilarities of presence/absence transformed data were used in the PERMANOVA tests to investigate the effects of ADD_0_, forest management intensity and study regions on beta-diversity community shifts for all three actors. PERMANOVA tests were calculated with the *adonis*() function in R package *vegan* and pairwise multi-level comparisons using the *pairwise.adonis2*() function in R package *pairwiseAdonis* [131]. In all PERMANOVA tests, piglet ID was indicated as a random factor by using the strata argument to constrain the permutations within each piglet cadaver. The model was specified in the same way as for alpha-diversity estimates.

For the alpha-diversity models of volatiles and flies, we used the data sampled across the whole decomposition period to gain results from the largest possible picture. For bacteria, we could only use samples from day 0, 2 and 4 after day 0 of exposure because, after day 4, the soft tissue of snouts was no longer detectable for taking accurate and comparable samples. Furthermore, we could only use fly samples with non-zero occurrences (e.g. no fly occurrences in progressed decay) in the beta-diversity models, because dissimilarity matrices cannot be calculated with zero-entries. Additional figures in the electronic supplementary material were created using the R package *ggplot2* [132].

#### 2.5.3. Interdependence of the three actors (bacteria, volatiles and flies)

In the second part of the analysis, we initially aimed to investigate the correlation of all three community compositions (bacteria, volatiles and flies) and then the temporal and spatial factors that might regulate the pairwise correlation strength. Here, we focused on the first few days of decomposition (data from days 0, 2 and 4) until the point at which bacterial mucosa swabs could no longer be sampled. For this, we first calculated non-metric multi-dimensional scaling (function *metaMDS*() in R package *vegan*) based on Bray–Curtis dissimilarity matrices by using Hellinger-transformed relative abundance (data from bacteria and volatiles for days 0, 2 and 4). The fly data matrix for sampling days 2, 4 and 6 was used for the comparisons of bacterial communities and fly occurrence on days 0, 2 and 4, because the pitfall traps were opened for each 48 h interval. For all three matrices, it is essential that row entries (= samples) are identical for each row number, and that matrices have the same length; this resulted in *N* = 208 rows in this analysis. Three-dimensional NMDS ordinations for bacteria, volatiles and flies were used in three pairwise Procrustes correlations, because three dimensions reflected variation in all three matrices better than in two dimensions (stress value < 0.2). Next, Procrustean randomization tests were performed with the NMDS axes scores (functions *procrustes*() with symmetric rotation and *protest*() for significance in R package *vegan*). We calculated three pairwise Procrustean correlations: bacteria versus volatiles, volatiles versus flies, and bacteria versus flies. A significant Procrustean correlation indicates interdependence of two tested biotic communities [133,134].

#### 2.5.4. Factors regulating interdependence between the actors (bacteria, volatiles and flies)

In the last step, we used Procrustean residuals (the residuals obtained from pairwise Procrustean correlations) as new response variables to explore the effects of ADD_0_, forest management intensity and study regions on the correlation strength of each Procrustean correlation [133]. Small residuals stand for large correlation strength (= strong interdependence) between two actors and vice versa. The variation in the pairwise Procrustean residuals was investigated using linear mixed effect models (function *lmer*() in R package *lme4*) and nonlinear GAMMs (functions *gam*() in R package *mgcv*), depending on the results of the diagnosis plots of the models. To test the significance of the effects of the covariates in the nonlinear model, we used analysis of deviance (*F*-test) (function *Anova*() in the R package *car* [135]). In the GAMMs, ADD_0_ was included as a nonlinear smoothing function. In all models, piglet ID was included as a random factor. The model structure was specified as described above: Procrustes residuals ∼ ADD_0_ + forest management + region + (random | piglet ID). The pairwise comparisons for the categorial variable region were calculated using the function *emmeans*() in the R package *emmeans*. The model validations and visualizations were performed as described above for alpha-diversity models.

#### 2.5.5. Correlation analysis at community level

Only in few cases could a direct functional link between a particular bacterium, a particular volatile and a particular necrophagous insect be determined experimentally. For example, specific methionine degrading bacterial taxa produce distinct sulfur-containing VOCs, such as dimethyl disulfide or dimethyl trisulfide (DMTS) [136–138]; these single volatiles mediate responses in necrophagous species such as the burying beetle *Nicrophorus vespilloides* (Coleoptera: Silphidae) [105,139] and gravid *Lucilia sericata* (Diptera: Calliphoridae) females [70]. In natural environments and *in situ* experiments, this is mostly not possible. Whole VOC bouquets, rather than single compounds, are obviously emitted from carrion sources [7], and these volatile blends can be produced by many different strains from the bacteria kingdom [2,31]. In addition, by using a next-generation sequencing approach, a whole plethora of bacterial SVs can be identified, but these might be taxonomically as yet unknown, and hence no functional knowledge is currently available. We therefore decided to analyse our data not at the level of particular bacterial or insect species or chemical compounds, but rather on a community level, as this nevertheless has the potential to reveal the mechanistic interdependent dynamics taking place during vertebrate decomposition at a holistic scale.

## 3. Results

Overall, across all sampling days, we recognized six stages of piglet decomposition: fresh, putrefaction, bloated, post-bloating, advanced decay and dry remains. The decomposition stages of individual carcasses were not of equal duration. For example, six cadavers reached the dry remains stage as early as 6 days post-mortem (fastest decomposition) and 13 cadavers reached the last decomposition stage on day 30 (slowest decomposition) (electronic supplementary material, figure S2).

### 3.1. Distribution of bacterial families, volatile organic compounds and fly species across sampling days

**Figure 1.**
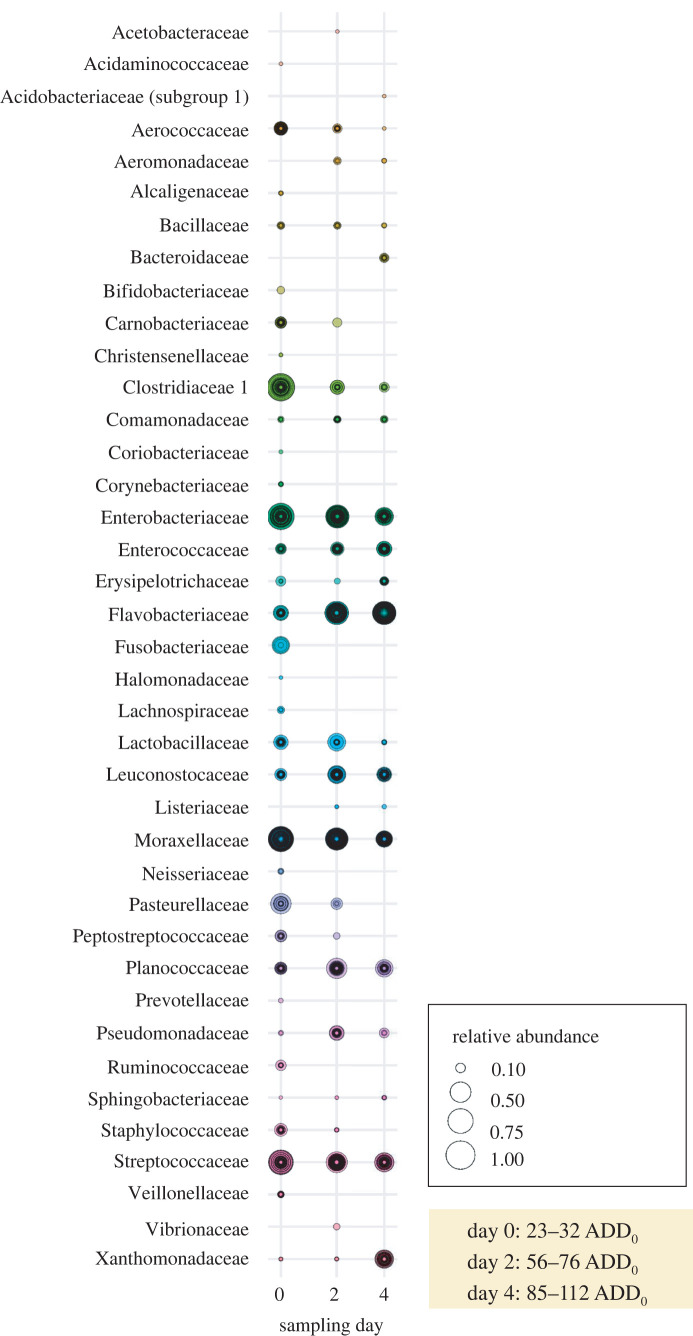
Relative abundance of bacterial taxa (agglomerated on family level, *N* = 39, greater than 1% abundance per family and, on average, 85.34% of all sequence reads; coloured gradation designates different bacterial families) within each sample obtained from snout swabs on the sampling days 0, 2 and 4 of piglet decomposition across all study regions and identified using 16S rRNA gene sequencing. ADD_0_ = accumulated degree-day.

After the raw sequence reads had been processed using QIIME2, the sample coverage values [101] per sample ranged from 0.999 to 1.0, indicating a very high level of sequence saturation (completeness). All further analyses were then performed with samples rarefied to a sample coverage of 0.999 each. In sum, 34 131 826 sequence reads remained with an average of 155 853 reads per sample. In total, 15 269 taxa at SV level were obtained, with an average of 691 per sample. We observed, at family level, 167 different taxon groups with a taxonomically valid family name; other taxa remained taxonomically not classified at family level. The taxa taxonomically classified at family level encompassed 29 263 875 sequence reads (85.7% of all reads and 54.5% of all SV) with an average of 51.7 families per sample (figure 1). Thus, a large majority of sequence reads were classifiable at family level.

**Figure 2.**
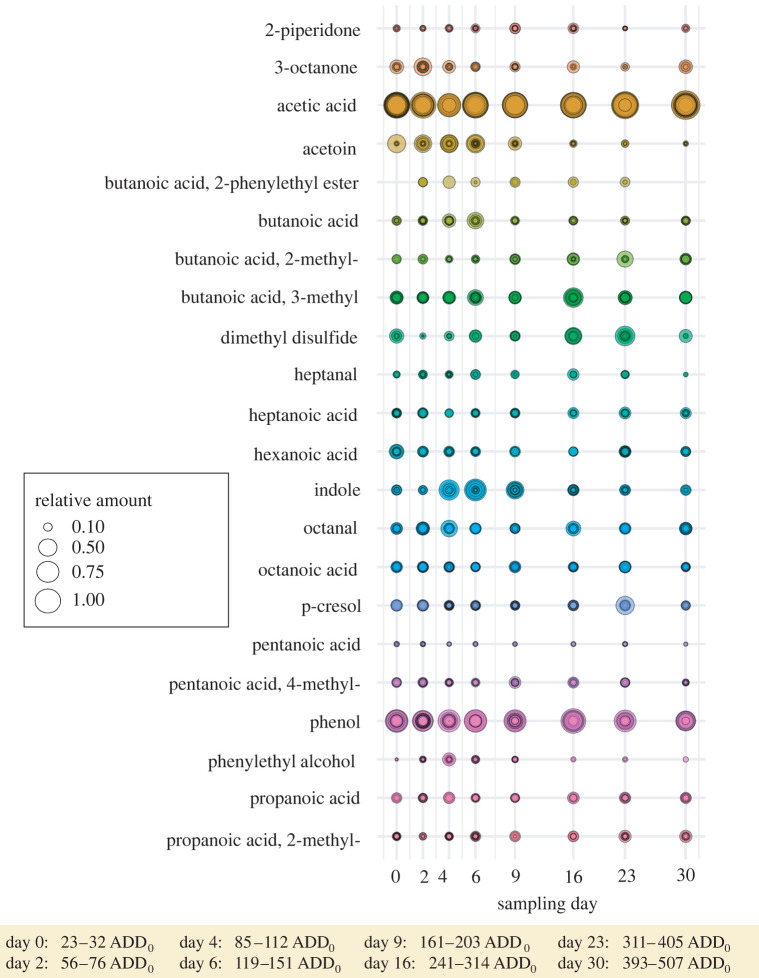
Relative amounts of 22 cadaveric VOCs (coloured gradation designates different compounds) that were identified within each sample and across all sampling days during decomposition and that were emitted from decomposing piglet cadavers across all study regions. ADD_0_ = accumulated degree-day.

Twenty-two VOCs that were emitted by piglet cadavers across all sampling days of decomposition were identified and validated (figure 2). Acetic acid and phenol were the most prominent compounds across all sampling days. For most of the VOCs, the emitted amounts did not change over sampling days, or they varied greatly between piglets; however, the amount of indole and phenylethyl alcohol, for example, obviously increased during the first days of decomposition.

**Figure 3.**
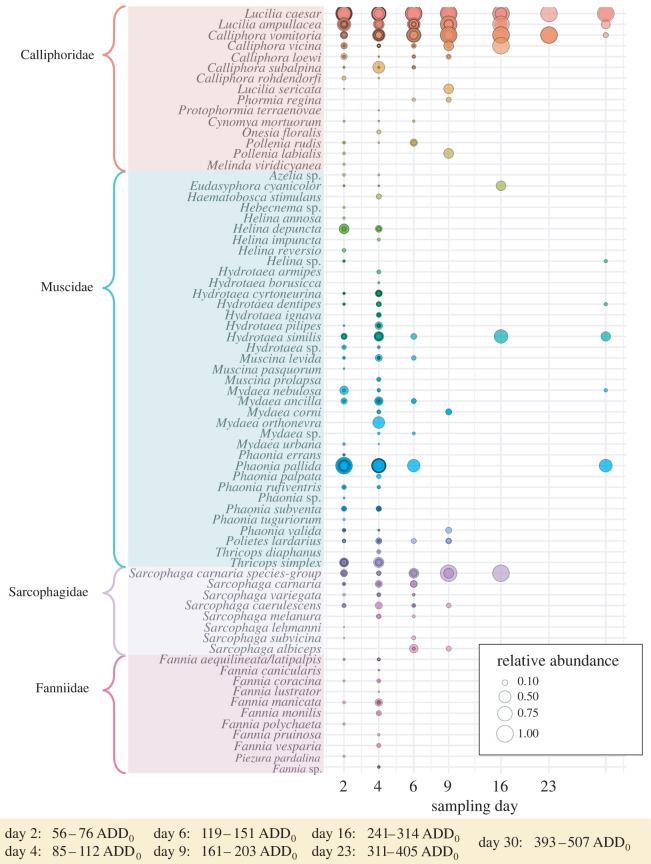
Relative abundance of sampled fly species (families Calliphoridae, Muscidae, Sarcophagidae and Fanniidae; coloured gradation designates different fly species in the associated fly families) within each sample and across all sampling days and study regions during piglet cadaver decomposition. Traps were first opened for cadaver exposure on day 0 for a 48 h interval and, afterwards, were opened each for 48 h before each sampling event. ADD_0_ = accumulated degree-day.

With regard to the trapped flies on carrion, 11 147 fly individuals belonging to four families (Diptera: Calliphoridae, Muscidae, Sarcophagidae and Fanniidae) were collected across the whole decomposition period (figure 3). The most abundant flies (greater than or equal to 55 individuals over all samples) were found from the following 10 taxa (families of the listed species are depicted in figure 3). *Lucilia caesar* (5996 ind.) was by far the most abundant fly, followed by *Phaonia pallida* (1693 ind.), *Lucilia ampullacea* (998 ind.), *Calliphora vomitoria* (937 ind.), *Hydrotaea similis* (449 ind.), *Thricops simplex* (298 ind.), *Hydrotaea dentipes* (66 ind.), *Helina depuncta* (60 ind.), *Hydrotaea cyrtoneurina* (55 ind.) and *Phaonia subventa* (55 ind.). Fifteen fly taxa (less than 55 and greater than 10 ind.) were considered as sub-dominant and 38 taxa (less than 10 ind.) as rare species. Eight taxa could only be determined to genus or species group level.

### 3.2. Factors shaping alpha- and beta-diversity of the three actors (bacteria, volatiles or flies)

An overview of the results for temporal and spatial factors regulating the alpha- and beta-diversity of the three actors is presented in table 1. More detailed information of the model results can be found in the electronic supplementary material, table S1.

**Table 1.** Overview of effects of ADD_0_ (accumulated degree-day), forest management, and study regions on alpha-diversity (total abundance and species richness) and beta-diversity (community composition and species turnover) estimates of the three actors (bacteria, volatiles and flies). For more detailed information of the model results, see electronic supplementary material, table S1. — = not applicable; n.s. = not significant; alpha-diversity models: GAMMs; bacteria *N* = 219, volatiles *N* = 549, flies *N* = 495; beta-diversity models: PERMANOVAs; bacteria *N* = 219, volatiles *N* = 549, flies *N* = 275; community composition: Bray–Curtis dissimilarity matrices on Hellinger-transformed relative abundance data; species turnover: Jaccard dissimilarity on presence–absence data; study regions: ALB = Schwäbische Alb, HAI = Hainich-Dün, and SCH = Schorfheide-Chorin.

actor	alpha-diversity		beta-diversity	
explanatory variables	total abundance	species richness	community composition	species turnover
bacteria				
ADD_0_	—	<0.001	<0.001	<0.001
forest management	—	n.s.	<0.001	<0.001
region	—	n.s.	n.s.	n.s.
volatiles				
ADD_0_	<0.001	<0.001	<0.001	<0.001
forest management	n.s.	n.s.	0.003	n.s.
region	n.s.	n.s.	n.s.	n.s.
flies				
ADD_0_	<0.001	<0.001	<0.001	<0.001
forest management	0.006	0.011	n.s.	n.s.
region	ALB-SCH: 0.024^a^	ALB-SCH: 0.035^a^	n.s.	n.s.

^a^Result obtained from the *summary*() function in R package *base* [121].

**Figure 4.**
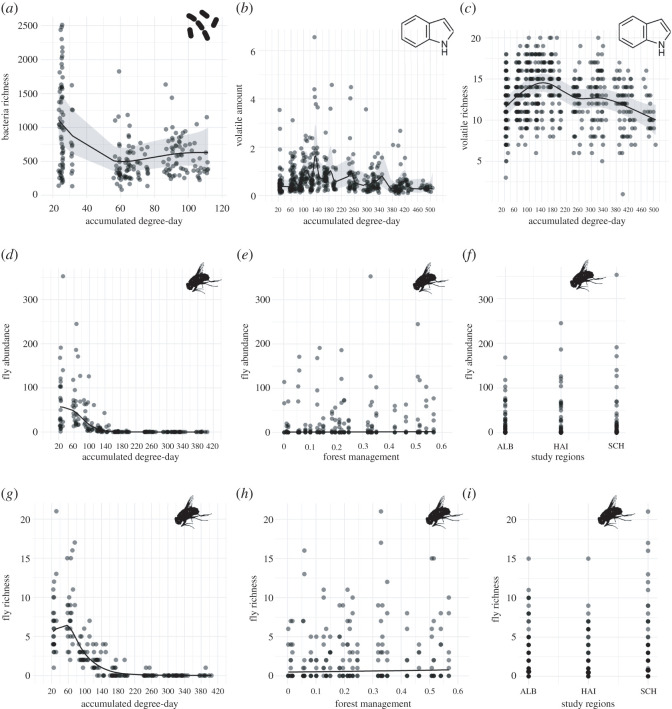
Marginal effects plots showing results of GAMMs. The following significant effects are displayed: the effect of (*a*) ADD_0_ (accumulated degree-day) on bacteria richness, (*b*) ADD_0_ on volatile amount, (*c*) ADD_0_ on volatile richness, (*d*) ADD_0_ on fly abundance of the fly families Calliphoridae, Muscidae, Sarcophagidae and Fanniidae, (*e*) increasing forest management intensity on fly abundance, (*f*) study regions on fly abundance, (*g*) ADD_0_ on fly richness, (*h*) increasing forest management intensity on fly richness and (*i*) study regions on fly richness. Bacteria, volatiles and flies were all sampled simultaneously on decomposing piglet cadavers. More detailed model result information is shown in the electronic supplementary material, table S1.

The GAMM fitted to the bacterial richness explained 34.5% (adjusted *R*^2^) of the observed variance. The temperature–time index ADD_0_ significantly affected the richness of bacteria (table 1; electronic supplementary material, table S1) and is plotted in figure 4*a*. Highest bacterial richness was detected at the start of decomposition (20–40 ADD_0_, day 0), then decreased until 60 ADD_0_ (day 2), slightly increased until 100 ADD_0_ (day 4) and stagnated between 100 and 120 ADD_0_ (day 4). Forest management and study regions did not affect the richness of bacteria. However, the community composition and species turnover of bacteria were influenced by ADD_0_ and forest management (table 1; electronic supplementary material, table S1). Most of the variance in species composition was explained by ADD_0_ (*R*^2^ = 12.3%) and the forest management intensity (*R*^2^ = 0.4%). ADD (*R*^2^ = 6.4%) and forest management intensity (*R*^2^ = 0.5%) best explained the variance in species turnover of bacteria, although the explained variances in bacteria beta-diversity estimates were relatively small.

Concerning the emitted VOCs from piglet cadavers, the GAMM fitted to the amounts and richness of volatiles explained 27.4% (adjusted *R*^2^ for total amount) and 15.3% (adjusted *R*^2^ for volatiles richness) of the observed variances. ADD significantly affected volatile amount and volatile richness (table 1; electronic supplementary material, table S1) and is displayed in figure 4*b*,*c*. The variation of total volatile emission between piglet individuals was high resulting in a relatively jittered regression curve in this model fit, although a global increase until 140 ADD_0_ (day 6) followed by a decrease until the end of decomposition was visible. An increase of volatile richness was also detected until 140 ADD_0_, followed by a decrease in volatile compounds until the end of decomposition. With regard to volatile composition and volatile turnover, the temperature–time index ADD_0_ also explained the variations (*R*^2^ of 0.7% for volatile composition and *R*^2^ of 0.8% for volatile richness) best (table 1; electronic supplementary material, table S1). In addition to the effect of ADD_0_, forest management intensity explained 0.2% of all variation in volatile composition, although the explained variances for volatile beta-diversity were low.

The GAMM fitted to the fly abundance explained 77.9% (adjusted *R*^2^) and the GAMM fitted to fly richness explained 89.3% (adjusted *R*^2^) of the observed variances. ADD_0_, forest management intensity and study regions affected both fly abundance and richness found on piglet cadavers across the whole decomposition period (table 1; electronic supplementary material, table S1). Fly abundance was highest between 20 and 30 ADD_0_ (day 0) but decreased until about 150 ADD_0_ (day 6) and was followed by a sharp break with only few fly occurrences from this point onward until the end of decomposition (figure 4*d*). However, fly richness first increased until about 60 ADD_0_ (day 2) followed by a decrease until about 200 ADD_0_ (day 9) and showed a similar breakpoint as for fly abundance with only a few fly species until the end of decomposition (figure 4*g*). Forest management intensity also affected fly abundance and richness found on carrion. More flies were found on forest sites that were managed more intensively (figure 4*e*); however, the positive slope was low. In agreement with the results of fly abundance, more fly species were found on highly managed forest sites (figure 4*h*). Furthermore, the highest abundance and richness of the four carrion-associated fly families were found in the study region SCH compared with the study region ALB (figure 4*f,i*). The temperature–time index ADD_0_ solely explained the variations in community composition and species turnover of carrion-associated flies, with an explained variance (*R*^2^) of 8.8% for community composition and 8.1% for species turnover (table 1; electronic supplementary material, table S1). Neither forest management nor the three study regions affected the two beta-diversity estimates.

Summarized, the temporal progression of decay (ADD_0_) explained most of the variation of the alpha- and beta-diversity estimates of epinecrotic bacteria, emitted volatiles and attracted flies. To a lower degree, forest management intensity shaped the beta-diversity of the bacterial and VOC profiles, and the alpha-diversity of attracted flies towards the carrion resource.

### 3.3. Interdependence of the three actors (bacteria, volatiles and flies)

**Figure 5.**
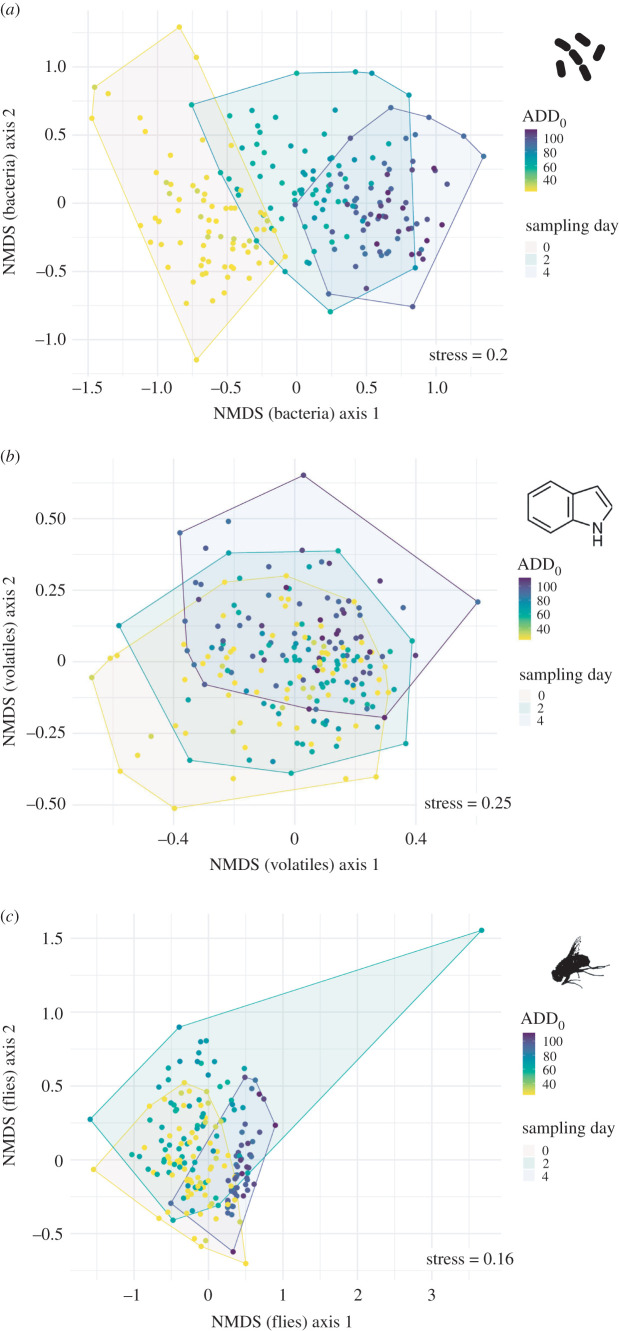
Two-dimensional NMDS ordination plots illustrating the differences between carrion-associated (*a*) bacteria, (*b*) volatiles and (*c*) fly communities based on the Bray–Curtis dissimilarities of the relative abundance and the temperature–time index ADD_0_. The three different sampling days are indicated with hulls (minimum convex polygons); *N* = 208 for all ordinations; ADD_0_ = accumulated degree-days.

Procrustes analyses revealed a strong correlation (= interdependence) between bacteria and volatile compositions (*m*^2^ = 0.948, *r* = 0.229, *p* = 0.001). The correlation was even stronger between bacteria and fly communities (*m*^2^ = 0.856, *r* = 0.379, *p* = 0.001). A lower but still significant correlation was found between volatiles and fly communities (*m*^2^ = 0.975, *r* = 0.157, *p* = 0.01) (figure 5).

In summary, a strong inter-kingdom relationship between bacteria, volatiles and flies could be revealed.

### 3.4. Factors regulating the interdependence between the actors (bacteria, volatiles and flies)

Procrustean residuals, the residuals obtained from the three pairwise Procrustes correlations, were used in linear and nonlinear regression analyses to investigate the temporal and spatial factors that possibly regulated the correlation strength (= interdependence) between the actors.

**Figure 6.**
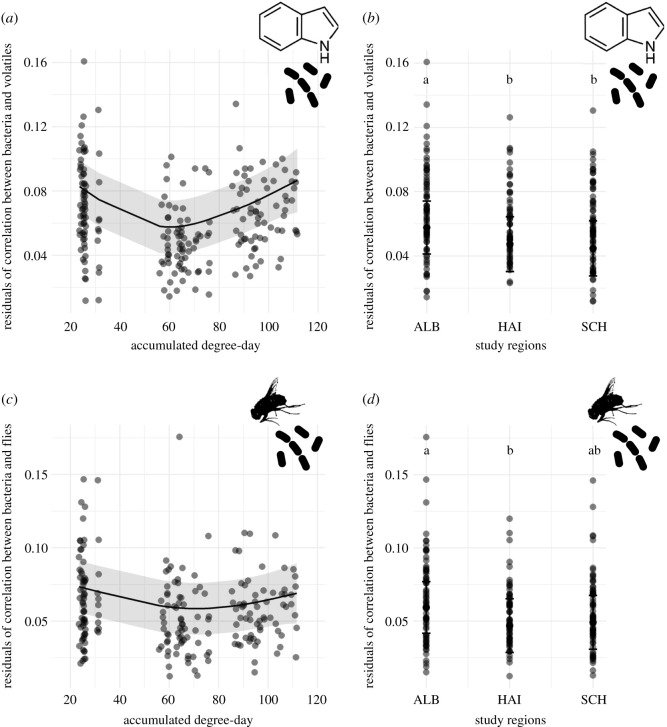
Marginal effects plots showing the results of the GAMMs. The following significant effects are displayed: the effect of (*a*) ADD_0_ (accumulated degree-day) and (*b*) study regions on the residuals of Procrustean correlation between bacteria and volatiles; the effects of (*c*) ADD_0_ and (*d*) study regions on the residuals of Procrustean correlation between bacteria and flies found on carrion. Small residuals indicate a large correlation strength or interdependence between two respective actors and vice versa. Different letters indicate significant pairwise differences. ALB = Schwäbische Alb, HAI = Hainich-Dün, SCH = Schorfheide-Chorin. More detailed information on the model results is shown in the electronic supplementary material, table S2.

The GAMM fitted to the residuals of the Procrustean correlation between bacteria and volatiles found on carrion explained 31% (adjusted *R*^2^) of the variance of the data and revealed that ADD_0_ and the study regions regulated this correlation (electronic supplementary material, table S2). The interdependent relationship fluctuated across ADD_0_, having the weakest correlation strength (= larger residuals) between 20 and 40 ADD_0_ (day 0). A stronger correlation (= smaller residuals) was then found between 55 and 80 ADD_0_ (day 2) and again a lower correlation between 80 and 120 ADD_0_ (day 4) (figure 6*a*). Furthermore, the lowest correlation was found in the study region ALB compared with the regions HAI and SCH (figure 6*b*). Forest management intensity did not affect the correlation strength between bacteria and volatiles (electronic supplementary material, table S2).

The GAMM fitted to the correlation between bacteria and flies explained 22.3% (adjusted *R*^2^) of the variance. As in the GAMM with regard to the residuals of bacteria and volatile correlation, ADD_0_ and the study regions influenced the correlation strength between bacteria and flies on piglet cadavers (electronic supplementary material, table S2). Correspondingly, the lowest correlation (= largest residuals) was found between 20 and 40 ADD_0_ (day 0). The correlation then increased (= residuals decreased) between 55 and 80 ADD_0_ (day 2) and subsequently slightly decreased again between 80 and 120 ADD_0_ (day 4) (figure 6*c*). This pattern remained nearly the same when considering only the data of each separate study region (electronic supplementary material, figure S7). In the region ALB, the lowest correlation between bacteria and volatiles (= larger residuals of Procrustean correlation) was found compared with the region HAI (figure 6*d*). Forest management did not affect the correlation strength between bacteria and volatiles. Furthermore, we found no significant effect of the temporal and spatial factors on the correlation strength between the emitted volatiles and the attracted flies in the linear model (electronic supplementary material, table S2).

In summary, the interdependence between bacteria and volatiles and between bacteria and flies was regulated by the temporal progression of decay and the three study regions but was stable across forest stands with increasing forestry activities.

## 4. Discussion

The current study shows that the temporal progression of decay, represented by ADD_0_, explains most of the variation of the alpha- and beta-diversity estimates of three actors, namely epinecrotic bacteria, emitted volatiles and attracted flies. To a lower degree, forest management intensity, as one spatial factor, shapes the beta-diversity of the bacterial and VOC profiles, and the alpha-diversity of attracted flies towards carrion. We have further revealed a strong inter-kingdom relationship between the three actors. The interdependence between bacteria and volatiles and between bacteria and flies is regulated by ADD_0_ and the three study regions but is stable across forest stands with increasing management activities.

### 4.1. Factors shaping alpha- and beta-diversity of the three actors (bacteria, volatiles or flies)

Ambient temperature is the one of the main environmental factors influencing all chemical and physiological reactions taking place during the terrestrial decomposition of animal tissue, and the activities of the carrion-associated actors (bacteria, volatiles and flies) are also highly temperature dependent [8,73]. We have shown that ADD_0_ mainly determines alpha- and beta-diversity estimates of all three actors. These results indicate a temporal successional shift for each investigated aspect during the progression of decay.

We have demonstrated highest bacterial richness at the start of decomposition, and with progressed decay, fluctuation with the main decrease in species richness until a point at which piglet snout mucous membranes are no longer detectable (after day 4). Further, bacterial community composition and species turnover also change with ADD_0_, with communities becoming more homogeneous over decomposition time. Carrion-associated bacteria are the main drivers initiating vertebrate decomposition [14,15]. At the start of the decay process, stillborn piglet cadavers host diverse epinecrotic microorganisms that are potentially derived from the mother sow (during passage through the birth canal [140]) and from the animal shed. As decomposition progresses, the proliferation and competition of diverse bacteria communities on the cadaver may result in a dominance of a few bacterial taxa with a significant change in community composition and species turnover [14,38]. Dangerfield *et al*. [141] and Harrison *et al*. [142] have also observed a decrease in carrion-associated bacteria diversity and a change to more similar bacteria communities over decomposition time. However, in a pilot study, Pascual *et al*. [19] report an increase in bacteria richness in the first few days of decomposition in cut snout samples of stillborn piglet cadavers. Interestingly, this discrepancy from the results in our study (figure 4*a*) is based on slightly different analytical procedures and the specific subset of only nine piglets (out of the 75 piglets used here) from Schorfheide-Chorin as used by Pascual *et al*. [19].

Vertebrate carrion emits the most intense smell during active decay when maggot masses feed on the burst carcass [46,47]. Accordingly, we have observed that the amount and number of VOCs, which are mainly emitted as by-products from endo- and epinecrotic bacteria, fluctuate with the temporal progression of decay during our study. Despite a high variation in the emitted volatile amounts from each individual carcass, indicated by a relatively jagged regression curve (figure 4*b*), a general bell-shaped curve progression is visible with a peak in volatile emission showing the most complex profile and the highest volatile amount at about 140 ADD_0_ (sampling day 6; post-bloating (= active decay) dominated; see electronic supplementary material, figure S2). Moreover, with the increasing ADD_0_, the emitted volatile composition and single compounds contribution change significantly revealing a changing pattern in the emitted odour profile that is seen across the decomposition process and that is most likely connected to the above-mentioned temporal change in bacteria community composition. Similarly, we have observed the maximum volatile emissions and number of compounds during the active (= post-bloating) decay of adult pig carcasses, after the body breaks up as a result of the overpressure of the microbially produced gases [27,46,47,103]. Furthermore, we have shown that the volatile blends do not change across the study regions indicating a spatially stable cadaver volatile emission pattern. Thus, cadavers seem to have a common decomposition-driven ‘smell’ across geographical regions, and the VOC profile is not significantly affected by spatial distance. This suggests that the chemical changes of vertebrate tissue are promoted primarily by the intrinsic temporal progression of decay [18].

Carrion-associated flies representing the families Calliphoridae, Muscidae, Sarcophagidae and Fanniidae are the primary insect colonizers of fresh carrion [78,143]. Thus, we have found highest abundance and species richness of these carrion-associated flies during early decomposition, and afterwards, the numbers decrease radically. Moreover, we have shown a significant change in fly community composition and species turnover across the decomposition progress. Species-specific successional arrival patterns within the four investigated fly families (e.g. community composition and succession in carrion flies in [53,144] or flesh flies in [145]) might explain the species turnover. Further analyses at species level should also help to interpret species-specific results and provide possible explanations for species turnover at the community level.

To a lesser degree than the effect of ADD_0_ on each separate actor, increasing forest management explains the variation in the community composition of epinecrotic bacteria and emitted volatile bouquets but not the variance in fly communities. The transmission of environmental bacteria from differently anthropogenically disturbed surroundings might occur as early as a few minutes after death, once the first bacteria-carrying insects colonize the cadaver, and thus might rapidly alter the initially dominating epinecrotic microbiome on a carcass [54,55]. Consequently, the volatile composition, mainly emitted by carrion-associated bacteria, also changes [7]. However, related to those changes, the attraction of fly communities towards the cadavers does not shift. To ensure their rapid and successful detection of a carcass resource, carrion-associated flies should thus have evolved as generalists colonizing all kinds of rotten substrate and therefore should respond to constantly available, common odour compounds as a reliable indicator of dead animal biomass [22].

Instead of having an impact on the community composition of flies, human activities in forests change the alpha-diversity of carrion-associated flies with a higher abundance and more fly species being attracted to the piglet cadavers in forest stands with a larger human impact. Scherber *et al*. [84] have found more individuals and more species of Diptera in German forests having a lower canopy cover. Since intensively used forests consist mainly of monocultures (e.g. pine trees) that also form a loose tree canopy, we expect that carrion-associated flies probably also benefit from open forests stands. In such forests, carrion volatiles can spread unimpededly, and thus the localization of the resource by carrion-associated flies can be assumed to be faster and more successful for more fly species. Nazni *et al*. [146] have shown that the flight range of the house fly *Musca domestica* (this species also visits outdoor exposed carrion; e.g. [51]) can be several kilometres (up to 7 km). Thus, the flight range of carrion-associated flies searching for resources might be larger than one unit of forest management, which is, especially in the study region Schwäbische Alb, small-scaled in some places. Such extended fly ranges of carrion-associated flies underline our finding that the attraction and, consequently, the assemblage of fly communities towards the cadavers do not shift significantly with a changing small-scaled forest management regime.

Moreover, we have found the highest abundance and richness of carrion-associated flies in the study region Schorfheide-Chorin. Compared with the regions Schwäbische Alb and Hainich-Dün, Schorfheide-Chorin in northeast Germany is outstanding as this region harbours by far the most abundant and diverse insect communities, as also emphasized by other arthropod studies conducted in the Biodiversity Exploratories project [82,147–149]. Formerly used as large hunting grounds for red deer [150], the forests in the region Schorfheide-Chorin are characterized by potentially high numbers of large vertebrate cadavers, resulting in a stable and relatively undisturbed carrion-associated insect community (for the high abundance of copronecrophagous dung beetles in the red deer area Schorfheide-Chorin, see von Hoermann *et al*. [149]), which might explain the highest fly abundance and richness in this study region. Moreover, extended pine tree stands only occur in this study region, which has low canopy cover as previously discussed.

### 4.2. Interdependence of the three actors (bacteria, volatiles and flies)

In terrestrial ecosystems, vertebrate carrion is a focal point for complex multi-species communities that interact with each other during the progression of decay [6], and the emission of VOCs is assumed to mediate the dynamic relationship between carrion-associated microorganisms and finally attracted insects [7]. In our study, epinecrotic bacteria, emitted volatiles and carrion-associated fly community compositions show a strong correlation, as revealed by the significant Procrustes test results that demonstrate the biotic multi-trophic interdependence taking place on vertebrate cadavers and strongly indicate the chemical attraction of carrion-associated flies by bacterial VOCs.

The lowest detected Procrustes correlation has been observed between the pair of volatiles and flies and can be explained because flies do not solely use olfactory stimuli to locate carrion but also employ visual and tactile cues that are also important for the inspection and, finally, establishment at the resource. Wall & Fisher [151] have shown that visual cues are, in addition to long-range olfactory cues, responsible for the selection of the final landing site over a short range in the early carrion colonizing calliphorid fly *Lucilia sericata*. Carrion-mimicking flowers also make use of the synergy between olfactory and visual stimuli by imitating the odour profile and visual appearance of a rotting substrate for the successful attraction of pollinators [152,153]. Moreover, the ovarian status of female flies can also affect their response to volatile cues [69], and some VOCs serve not only as attractants, but also as repellents [70]. The lowest correlation between the examined volatiles and fly matrices might also be a consequence of the fact that not all odour compounds emitted by carrion elicit a biologically active response in carrion-associated insects. Studies involving gas chromatography coupled with electroantennographic detection improve our understanding of the physiologically active core volatiles that can be perceived by carrion-associated flies, thereby inducing a reaction and/or behavioural response in the examined insect antennae at the species level [154]. Furthermore, as we have been unable to verify all sampled compounds from our reference list, the data matrix might have gaps. For example, Zito *et al*. [155] have shown a clear preference of distinct carrion-associated flies (such as *Lucilia caesar* and *Calliphora vicina*) towards DMTS. This compound was however detected in our collected cadaver volatile samples in too small amounts and in too low numbers for useful analysis when using the Chromatoprobe filter system. However, von Hoermann *et al*. [105] have detected DMTS frequently in volatile samples emitted from piglet cadavers that were placed in oven bags and sampled using the dynamic headspace procedure with the Super-Q filter system and a 4 h sampling interval. Therefore, the use of different adsorbents might influence the VOCs that are identified in headspace sampling. For increasing the performance, Vass *et al*. [156,157] used triple-sorbent traps for the analyses of buried human remains.

The direct correlation between bacteria and volatiles was weaker than the indirect correlation between bacteria and flies (indirect = cadaver volatiles as mediators). We assume that this is attributable to additional volatile sources besides those originating from epinecrotic bacterial activity and that these additional volatiles were not captured in our analyses. Immature insects and mature scavengers can also release own volatile bouquets [43,45] that might also lead to behavioural responses in arriving flies. The indirect relationship between bacteria and flies was strongest in comparison with the direct relationship between bacteria and volatiles and between volatiles and flies, demonstrating a significant indirect inter-kingdom interdependence between epinecrotic bacteria and colonizing flies at vertebrate cadavers. Jordan & Tomberlin [8] have described, in their review paper, various biotic and abiotic interactions between carrion-associated bacteria and colonizing insects on vertebrate cadavers and have emphasized the mechanistic role of bacteria in the mediation of the insect colonization, as indeed we have emphasized with our large-scale study results. However, the link between the surface bacteria that are transmitted by flies to the decomposing microbiome [55] might have a stronger effect on the investigated correlation than the effect of the bacteria-derived volatiles on the attraction of flies. This further suggests that the dependence between surface bacteria and attracted flies is not uni-directional (bacteria are influencing flies) but bi-directional (flies also influence bacteria), an aspect that might therefore explain the highest correlation between these two actors (fig. 2 in [3]). As an alternative explanation, the comparatively weak correlation between the bacteria dataset and volatiles might arise from our focus on target compounds regarding the VOC profiles. Several VOCs that were released by the decomposing piglets might not be part of the target compound list, resulting in a decrease of VOC diversity in our analyses, whereas no similar reduction was applied on the bacteria dataset. Furthermore, bidimensional gas chromatography as successfully performed in cadaveric VOC analyses [103,158] could additionally help to increase the correlation between bacteria and volatiles. The problem of compound coelution in unidimensional gas chromatography can be diminished by a better resolution and consequently a higher cadaveric compound diversity in the two-dimensional GCxGC technique [103,159].

One possible example of a typical core volatile explaining the inter-kingdom communication between bacteria and flies is indole [160]. In accordance with our presented results on VOC emissions (figure 2), we can confirm the observation by Pascual *et al*. [19] of a strong increase of emitted indole that occurs during the first few days of vertebrate decomposition and that follows the abundance pattern mainly of the bacterial families Flavobacteriaceae, Xanthomonadaceae and Enterococcaceae [19] (figure 1). Indole can, in turn, be perceived by the carrion-associated blowfly *C. vicina* [153], and other blowflies such as *Lucilia sericata* [48] can thus explain the chemical fly attraction (figure 3) towards altered carrion resources at the species and compound level. However, further examples and explanations for such three-level interactions between bacteria, VOCs, and flies are extremely limited.

Compounds like indole and phenylethyl alcohol (2-phenylethanol), with both increasing during the first days of decomposition (figure 2), are very common during animal biomass decay [18,103,105]. Furthermore, in the previous paragraph mentioned bacteria like Flavobacteriaceae, Xanthomonadaceae and Enterococcaceae are very common representatives of the epinecrotic communities [2,13,19,141] and are important taxa for assessing overall bacterial diversity. For instance, regarding the impact of vertebrate scavengers on overall decomposition processes and consumer diversity [2], Dangerfield *et al*. [141] found no significant differences in microbial communities (including Flavobacteriaceae, Xanthomonadaceae and Enterococcaceae) of carcasses that experienced large amounts of scavenging activity when compared to carrion that observed very little scavenging activity (like the piglet carcasses in our study that were sheltered by wire cages).

### 4.3. Factors regulating the interdependence between the actors (bacteria, volatiles and flies)

The analysis of abiotic factors that possibly regulate the inter-kingdom interdependence between the actors demonstrates that both temporal and spatial factors (ADD_0_ and the study regions) modify the correlation strength between bacteria and volatiles and between bacteria and flies, but not the intensity of forest management. We have shown that, at the start of decomposition, piglet cadavers host a species-rich epinecrotic bacteria community that fluctuates over time and develops into a more homogeneous community with a lower richness of different bacterial taxa dominating the cadaver. Connected to the dominance of a few bacterial taxa, their emitted volatile blends possibly become less diffuse and evolve into a more consistent and stronger volatile signal over time. Thus, the interdependence between bacteria and attracted flies is tightest on decomposition day 2 (between 60 and 80 ADD_0_). However, with increasing ADD_0_, other volatile sources, such as those from fly larval secretions or other adult scavengers, come into play, and therefore the strength of the correlation between bacteria and volatiles becomes looser. This suggests that the mechanisms linking bacteria and emitted volatile blends and linking bacteria and fly communities shift mainly at a temporal scale (compare [15] where the bacterial activity is affected by temporal insect access or exclusion). However, the interdependent relationship between bacteria and volatiles and between bacteria and flies also differs at the spatial scale by having the lowest interdependence between the actors in the study region Schwäbische Alb. This implies that the biotic inter-kingdom interactions occurring on vertebrate cadavers are also habitat dependent [12,20]. The stated lowest interdependence of community assemblies at the inter-kingdom scale in the study region Schwäbische Alb can be explained by the lowest attraction of fly individuals and species towards the cadavers in this region (figure 4*f*,*i*) and demonstrates the importance of species-rich communities for increasing stability at the inter-kingdom scale. Taking the aspects together, one can see not only the main temporal mismatch of the inter-kingdom interactions that takes place during vertebrate decay, but also the spatial mismatch at the landscape level. The interdependence between epinecrotic bacteria, emitted VOCs, and attracted flies remains stable across increasing forest management indicating that human management intensity has no significant contribution to the disruption of the undisturbed chemical communication between bacteria and flies and, thus, potentially has no impact on the fly-dominated progress of decomposition in the differently managed terrestrial ecosystems.

### 4.4. Methodological restrictions and future studies

Perrault *et al*. [47] have discussed the methodological challenges that are faced when sampling decomposition VOCs at carrion sites. They have found markedly different trends in detected decomposition VOCs occurring at the different decomposition stages, highlighting the potential effects of weather variables such as moisture accumulation through rainfall with regard to the sampling device. Kasper *et al*. [161] have shown that environmental conditions modify volatile emissions. We have also found a high variation in the emitted VOCs and a relatively low explained variance in our models, both being connected to the decomposition VOC dataset. Thus, predictive modelling with VOC data from the field remains challenging. However, the moisture content of the cadaver tissue and the ambient humidity in close proximity to the carcass might help to explain some unknown variances in our models since moisture, in addition to temperature, represents one of the most important abiotic variables affecting decomposition progression and biotic activities at the carrion resource [40,162]. We suggest that future studies should include the measurement of tissue moisture content in order to verify the VOC profiles and to gain a better explanation for variations in the respective datasets. Furthermore, the VOC sampling approach using the Chromatoprobe filter system has the advantage of gaining quantitatively numerous cadaver volatile samples in a relatively short time, but at the expense of the quality of VOC composition. The missing VOCs (as discussed for DMTS) might explain some unknown variances in our model results. A different sampling approach by using the Super-Q technique described in von Hoermann *et al*. [105] might increase the quality of the data by verifying a higher number of sampled compounds. However, the invasive handling procedure over a long sampling time (piglets in oven bags over 4 h) would impact the decomposition process of the cadavers by altering the metabolic processes of different maggot species and the microbiome (bacteria as well as fungi). Finally, from a more analytical approach, one should keep in mind the highly innovative two-dimensional GCxGC-TOFMS (time-of-flight mass spectrometry) technique having the potential to reveal plenty of additional cadaveric VOCs. This was impressively demonstrated in the work of Dekeirsschieter *et al*. [103], where 832 VOCs released by a decaying pig carcass could be identified.

## 5. Conclusion

Our multi-trophic-level perspective on the interactions within local carrion-associated communities strongly suggests a strong interdependent relationship between epinecrotic bacteria communities, emitted VOC blends and attracted carrion-associated fly communities. However, ADD_0_ and the study regions, as two abiotic factors, mainly modified this interdependence revealing regulation at the temporal and spatial scales, although the interdependence between the actors is stable across forests with increasing management. Our results demonstrate not only a dynamic bacterial change over the progression of decomposition (represented by the temperature–time index ADD_0_) and the forest management gradient, but also stability between spatially distinct regions. Similarly, volatile emissions at piglet cadavers are dynamic across ADD_0_ and the forest management gradient and stable across regions. However, fly occurrence on piglet cadavers is dynamic across both space and time. Better knowledge of the holistic mechanisms that regulate community dynamics and inter-kingdom interactions taking place during vertebrate decay under natural conditions and across large spatial distances extend beyond a mere understanding of local biotic community structures and separate observations of biotic variations. By using a multi-taxa and large-scale carrion ecological approach, we have disentangled and emphasized the cascading importance of multi-trophic level, biotic interactions and their influencing abiotic drivers at temporal and spatial scales. Such results have implications for the comprehension of variations in decomposition rates, carrion food web dynamics and entire ecosystem functioning in terrestrial ecosystems [2,3,8,12,56,88].

## Data Availability

Data are stored in the BExIS database of the Biodiversity Exploratories project (https://www.bexis.uni-jena.de, datasets ID 31028, ID 31031 and ID 31032). The data are provided in the electronic supplementary material [163].
